# Potential Use of* Euphorbia hirta* for Dengue: A Systematic Review of Scientific Evidence

**DOI:** 10.1155/2018/2048530

**Published:** 2018-04-16

**Authors:** Sashini D. Perera, Uthpala A. Jayawardena, Chanika D. Jayasinghe

**Affiliations:** Department of Zoology, Faculty of Natural Sciences, The Open University of Sri Lanka, Nawala, Nugegoda, Sri Lanka

## Abstract

*Euphorbia hirta* commonly known as* Tawa-Tawa* is a plant used in folklore medicine in the Philippines for the treatment of dengue. Though,* E. hirta* has been extensively investigated for numerous bioactivities, limited studies have been conducted on the antidengue activity. Thus, the present study provides a comprehensive review of studies conducted on the antidengue activity of* E. hirta*. A systematic literature survey was carried out in scientific databases, PubMed®, Scopus, and Google Scholar, for research carried on the antidengue activity of* E. hirta*. The literature search identified a total of 867 articles: databases PubMed = 6, Scopus SciVerse® = 423, and Google Scholar = 437; one additional article was identified by searching reference lists. Eight full papers were entitled to the review; out of those, two studies focused on ethnobotanical surveys, three on animal experiments, one on human trial, and two on* in vitro* antiviral activities, and one was computational study. The available evidence conclusively demonstrates the potential of* E. hirta* against dengue as it holds significant antiviral and platelet increasing activities. However, the number of studies conducted to validate its antidengue activity was found to be inadequate. Hence, well-controlled clinical trials and contemporary pharmacological approaches including activity guided fractionation and elucidation of the mode of action are encouraged to establish the use of* E. hirta* for dengue.

## 1. Introduction

Dengue is a viral disease that impose the greatest human and economic burden in tropical and subtropical regions [1]. Dengue is caused by four 4 distinct, yet closely related, serotypes (DEN-1, DEN-2, DEN-3, and DEN-4) belonging to family Flaviviridae, genus* Flavivirus *[1]. It is transmitted to humans by* Aedes* mosquitoes, mainly* Aedes aegypti*, an urban breeding mosquito [1].

Dengue is endemic in over 100 countries including Asia, the Pacific, the Americas, Africa, and the Caribbean [2]. In recent decades, the incidences of dengue have increased rapidly, currently reaching pandemic levels [3]. It is estimated that around 50 million cases occur each year and more than 2.5 billion of people are at risk of infection [3]. The disease not only is onerous to healthcare but also adversely impacts the economy caused by illness, premature death, and increased healthcare costs [4].

Dengue possesses a wide clinical manifestation ranging from nonsevere to severe forms [5]. The symptoms of dengue range from a mild flu-like syndrome (known as dengue fever [DF]) to the most severe forms of the disease, which are characterized by coagulopathy, increased vascular permeability, and plasma leakage (dengue hemorrhagic fever [DHF]) which eventually leads to dengue shock syndrome (DS) [5]. Dengue associated mortality is usually reflected in the increase in the progression of DF patients developing DHF/DS [6].

The severity of dengue is amplified by the lack of effective treatment [7]. Usually, the clinical symptoms of dengue are managed through fluid balance, a supplement of electrolytes, and blood clotting parameters [8]. Anti-D immune globulin therapy in some instances is used for severe thrombocytopenia [9], but this is expensive making it challenging for developing nations to afford such a treatment regimen. The first dengue vaccine, Dengvaxia, has been licensed in 2015 for clinical practice [10]. However, this may not ensure the protection from infection of all serotypes [7].

Along with the allopathic care, most people living in tropical and subtropical countries depend on folk/traditional medicine to alleviate dengue infection; in fact herbal medicines have been invaluable therapeutic agents going back to the earliest human civilizations [7]. Currently, there is a resurgent interest in herbal medicine among the public considering its safety and cost-effectiveness. There have been approximately 30 different plant species found to have the potential to combat dengue, such as* Andrographis paniculata* [Hempedu Bumi (Malaysia)],* Alternanthera philoxeroides* (alligator weed),* Carica papaya *(papaya),* Cladosiphon okamuranus *(brown seaweed),* and Momordica charantia *[bitter melon, Peria (Malaysia)] [7]. Among these,* C. papaya* leaf juice has been widely used as remedy against dengue in many Asian countries [11–13]. In this regard, extensive research has been carried out to establish the platelet activating [13], white blood cell increasing properties [14], and membrane stabilization [15] potential of* C. papaya* leaf extracts.

*Euphorbia hirta* is another plant used in folk medicine to cure dengue fever by people in rural areas of the Philippines [7]. The leaves of* E. hirta*, locally known as “*Tawa-Tawa*” or* gatas–gatas*, are used to make a decoction that believe to alleviate viral infection and associated fever symptoms [7].* E. hirta* belonging to Euphorbiaceae family is a hairy herb grown in open grasslands, roadsides, and pathways. It is a popular folkloric plant in other countries like India, Sri Lanka, Malaysia, Java, and Vietnam [16]. Previously, the antiviral, antibacterial, antimalarial, antifungal, anti-inflammatory, anthelmintic, and antitumor properties of the plant have been validated [16]. Particularly for dengue, a commercial formulation (capsule) has been developed and the local communities in the Philippines consume it as a treatment for dengue [17].

Despite the popularity of* E. hirta* as a folk remedy for dengue, few scientific validations have been carried out. Therefore, the present review intends to provide a comprehensive account of available scientific evidence to validate the effectiveness of* E. hirta *against dengue. It is anticipated that this work will provide the platform for future investigations on* E. hirta*. Further, this attempt warrants exploring the bioactive compounds and possible mechanisms of action and will pave the way to develop a new antidengue drug lead from* E. hirta.*

## 2. Methodology

### 2.1. Search Strategy

A systematic review of published studies on the use of* Euphorbia hirta* against dengue was undertaken in accordance with the PRISMA (Preferred Reporting Items for Systematic reviews and Meta-Analyses) [18]. A comprehensive search of the literature was conducted in the following databases: PubMed (US National Library of Medicine, USA), SciVerse Scopus (Elsevier Properties SA, USA), and Google Scholar for studies published before 31 October 2017. The following medical subject headings and keywords, “*Euphorbia hirta dengue*”, “*Tawa Tawa dengue*”, “*gatas–gatas dengue*”, were included in the search. Results were limited to studies in English, while conference proceedings and commentaries were excluded.

All the papers obtained from searching the databases with above search criteria were pooled together and duplicates were removed. The remaining articles were initially screened by reading the “title” and thereafter the “abstracts.” Studies not satisfying the inclusion criteria (Section 2.2) were excluded at these stages. The remaining articles were screened in the final stage by reading the full-text and those not meeting inclusion criteria were excluded. Additional articles were obtained manually using the reference lists of included articles.

### 2.2. Inclusion/Exclusion Criteria

The following inclusion criteria were used: (a) ethnobotanical studies based on use of* E. hirta *against dengue, (b)* in vivo* studies investigating the platelet increasing, white blood cell increasing and membrane stabilization potential of* E. hirta*, tested on laboratory animals such as mice, rats, and rabbits, (c)* in vitro* cell culture studies conducted against dengue viruses, (d) molecular docking studies involving the interaction between phytochemicals of* E. hirta *and dengue virus proteins, and (e) studies published before 31 October 2017.

Studies were excluded based on the following exclusion criteria: (a) different species of* Euphorbia*, (b) other bioactivities other than dengue or related pathologies, and (c) reviews written on* E. hirta.*

## 3. Results

**Figure 1:**
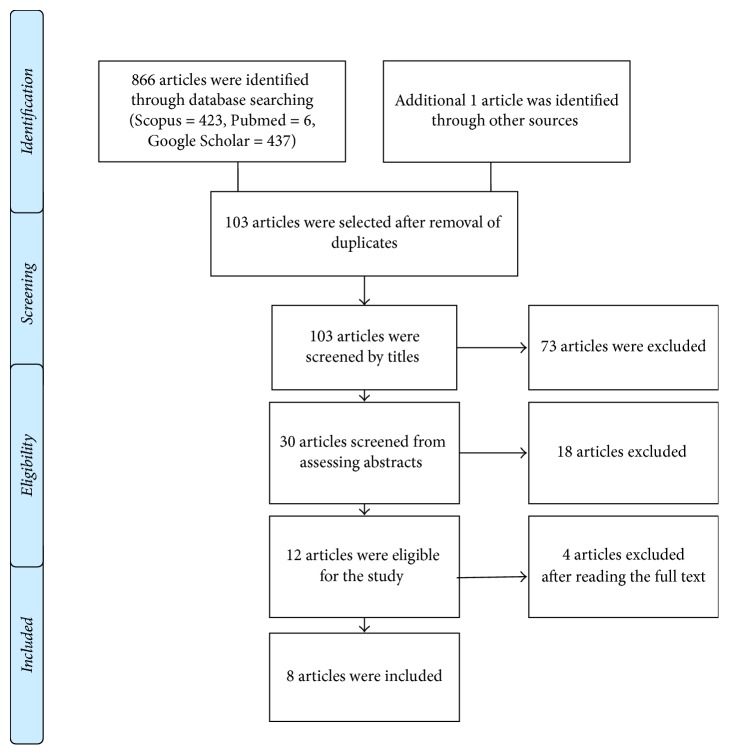
Schematic diagram representing the search strategy.

The literature search identified the following number of articles in the respective databases: PubMed (*n* = 6) and SciVerse Scopus (*n* = 423), and Google Scholar (*n* = 437). One additional article was identified by manually searching the reference lists and forward citations of included papers. After removing duplicates, the total number of articles included in the present review was eight.  Figure 1 presents the search strategy used in selecting the articles.

This systematic review pools the available scientific data on the effectiveness of* E. hirta* on dengue infection. Out of 8 studies, two studies were ethnobotanical surveys, 3 were animal experimentations, 1 study was from human trials, 2 were* in vitro* antidengue/antiviral studies, and 1 was from computations modelling (Table 1).

### 3.1. Ethnobotanical Survey

The two ethnobotanical surveys conducted on* E. hirta* have been carried out in different parts of the Philippines. A survey conducted in 3 different indigenous communities in the island municipality of Anda, Mt. Balungao, and Mt. Colisao municipal park found that mostly females 60–80 years of age with primary and secondary education generally use* E. hirta* extract to alleviate the symptoms of dengue and related bleeding episodes [19]. It was found that both topical application and oral treatment are recommended for symptom management of dengue [19]. Decoctions of leaves and bark are popularly administrated for dengue [19].

According to a descriptive survey conducted using questionnaires in Agoo, La Union, the Philippines, out of several plant species (Tawa-Tawa, Papaya, and Malunggay), Tawa-Tawa/*E. hirta* was the most popular medicinal plants consumed by patients who suffered from dengue fever [20].

### 3.2. Platelet Augmentation Activity

#### 3.2.1. Animal Models

Three studies have been conducted to investigate the platelet augmentation potential of* E. hirta. *All of them have established a significant platelet increasing activity following the administration of* E. hirta *leaf extract/decoction. These three studies have employed thrombocytopenic agents to lower the platelet counts prior to the administration of* E. hirta* extracts/juice.

Apostol et al. [21] had used ethanol (intraperitoneal injection) induced thrombocytopenic Sprague-Dawley rats to evaluate the platelet increasing activity of* E. hirta*. The results revealed that fourteen-day administration of 100 mg/kg of the lyophilized decoction of* E. hirta* had significantly increased platelet counts in rats [21]. Similarly, the bleeding time which was increased by the i.p. injection of ethanol was lowered by the* E. hirta* decoction. Simultaneously, the clotting time was decreased in* E. hirta*-treated rats compared to that of ethanol-induced thrombocytopenic rats [21].

A significant increase of mean platelet count was also observed following the oral treatment for 9 days of water extract of* E. hirta *leaves in Sprague-Dawley rats who were orally treated with Anagrelide (given for 15 days) for the induction of thrombocytopenia [22].

Similarly, in another study, when the expressed juice of* E. hirta *was administered in thrombocytopenic rabbit (by aspirin oral treatment), platelet counts were markedly increased after 24 hours [20].

#### 3.2.2. Clinical Study

A clinical study conducted with dengue patients (both age groups 30–55 and 14–25) admitted to the hospital Sir Ganga Ram Hospital Lahore showed that the oral treatment with herbal water of* E. hirta* had increased the platelet and total leukocyte counts after 24 hours. A significant platelet increase was also observed in the patients of 30–55 age group following the treatment with* E. hirta*, while the increment was not significant in the 14–25 age group, compared with the control group [23]. Similarly, the decrease in hematocrit values was not significant. Moreover, 70% patients had recovered from fever and flu-like symptoms [23].

### 3.3. Antidengue/Antiviral Activity

According to Tayone et al. [24], the ethyl acetate fraction of the whole plant of* E. hirta *extracted using methanol and dichloromethane significantly reduced (85%) the plaque forming capacity of dengue virus serotype 1 from ~1400 to ~200 PFU. Further purification of ethyl acetate fraction revealed the presence of 9 compounds. It was assumed that these compounds individually or synergistically may have contributed to antidengue activity [24].

Similarly, in another study, the ethanol extract of* E. hirta* demonstrated 34.7% inhibition of virus serotype 2 (DENV-2) under* in vitro* conditions [25].

### 3.4. Computer Modelling: Molecular Docking

Phytochemicals found in* E. hirta*, specifically quercetin, myricetin, rutin, kaempferol, gallic acid and protocatechuic acid, were used for docking study using Maestro (Glide) and Lead IT (FlexX) software applications. The dengue protease (2FOM) and dengue methyl transferase (2P40) have been selected as targets for binding assays. Out of the phytochemicals, quercetin exhibited the strongest binding with dengue targets. Hence, this study indicates that the* E. hirta *is effective against dengue virus [26].

## 4. Discussion

*Euphorbia hirta* is one of the most widely used medicinal plant in the Philippines as a treatment for dengue [19]. The ethnobotanical survey conducted in Pangasinan where dengue continues to be prevalent revealed most people, especially women, used this preparation as a supportive therapy for dengue [19]. Moreover,* E. hirta* was identified as the widely consumed remedy taken by dengue patients in Agoo, La Union [20]. These indicated the folkloric belief of* E. hirta* as a treatment against dengue. It was stated that both topical applications and oral treatments are recommended in traditional practice [20]. Particularly, the decoctions of leaves and aerial parts of the plant are commonly used for preparation. The ready availability of* E. hirta* in home gardens has increased its use as a home remedy [20].

Though* E. hirta* is used as a folkloric plant in India [16], Pakistan [27], and Sri Lanka [28] for common ailments (fever and infection), no studies have been carried out in these countries to investigate its use against dengue. Hence, more ethnobotanical surveys are warranted to overly validate the effectiveness of* E. hirta *as an antidengue medicinal plant in other parts of the world.

Three animal studies conducted using rats and rabbits established that the subacute administration of* E. hirta *leaves/whole plant increases the platelet counts [20–22].* E. hirta *has significantly increased the platelet counts in rats treated with ethanol to induce thrombocytopenia [21]. Intraperitoneal injection of ethanol may cause portal hypertension and hypersplenism leading to thrombocytopenia. Hence, the platelet increment by the* E. hirta* is allied to replenishment of platelet from the spleen. Furthermore, reduction of bleeding time in treated rats, compared to that of control rats, indicates an increase of platelet counts [21]. This reduction in bleeding time by* E. hirta* would be advantageous in managing DHF with the severity of bleeding episodes. Further, this study established an alteration of clotting time by* E. hirta* in treated rats compared with the control rats. This indicates the potential of modulating the coagulation pathways. Collectively, the hemostatic potential of* E. hirta* was validated by this study and these properties are prudent in managing dengue infection.

Coloma et al. [20] reported significant platelet augmentation in thrombocytopenic rabbits by* E. hirta *following 24 hours of treatment. However, this comparative analysis revealed that the platelet augmentation potential of* E. hirta* was lower than* Carica papaya* leaf extract [20].

*E. hirta* was also effective in increasing platelet counts in dengue patients of 30–55 age group [23]. Importantly, in around 70% of patients, flu-like symptoms were markedly reduced following* E. hirta* treatment which was attributed to the anti-inflammatory properties of the plant [23, 29]. Hence, in-depth analysis of immunomodulatory properties is fortified to establish the potential* E. hirta* on the alleviation of clinical symptoms of dengue. Since this study has enrolled patients only during the initial 72 hours of illness, additional prospective cohort studies are warranted to establish the clinical utility of* E. hirta* [23].

The phytochemical analysis of* E. hirta* has demonstrated the presence of alkaloids, carbohydrates, glycosides, saponins, phytosterols, phenolic compounds, and flavonoids [21]. Phenolics and flavonoids are well-known compounds with platelet increasing potential [21]. Even though* E. hirta* has a small amount of phenolic compounds compared to the other plants used for dengue (e.g.,* Carica papaya*), it may be sufficient to exert an effect promoting quality and quantity of platelets. If the phenolic compounds contain sufficient hydroxyls and another group (such as carboxyl) to form strong complexes-forming properties of tannins, then that could exert a positive effect in the platelet count in blood [20]. Moreover, flavonoids can improve megakaryocytes to produce sufficient numbers of platelets and to modulate the platelet counts [20]. In addition, it was assumed that the anti-inflammatory potential of flavonoids may have contributed to the alleviation of flu-like symptoms in dengue patients following the treatment with* E. hirta* [23].

Although* C. papaya* is more effective in platelet increasing activity, the antidengue potential of the plant is less established.* C. papaya* has exhibited moderate or low inhibitory action against DENV2 growth in* in vitro* conditions [30]. Conversely, the ethanol extract of* E. hirta *under* in vitro *condition demonstrated an 85% inhibition of plaque forming capacity of dengue virus serotype 1 (DENV-1) [24] and 34.7% inhibition of virus serotype 2 (DENV-2) [25]. Further purification of ethyl acetate fractions of the plant revealed 9 compounds which may exert an antidengue effect individually or synergistically [24]. The molecular docking studies using these compounds, the antidengue activity of* E. hirta* could be attributed to quercetin as it showed a stronger affinity towards the dengue protease (2FOM) and dengue methyl transferase (2P40) [26]. However, antidengue/antiviral assays should be performed on these pure compounds to validate these theoretical investigations.

According to the present survey, the existing evidence supports the platelet increasing and antiviral activity of* E. hirta* extracts. However, these studies are inadequate to establish a clinical use of the plant. Particularly, the mechanisms of platelet increasing activity have to be identified. Further, the effect of* E. hirta* on the vascular leakage, which is the main pathological indicator of severe dengue [1], may be corroborated to justify its utility in severe dengue conditions.

Herbal medicines have a prudent use in dengue as a supportive therapy as effective antiviral drugs and vaccines for all stereotypes have not yet been developed. There are several commercial herbal formulations including* C. papaya* leaf capsule and* Tawa-Tawa *capsule in the market as over-the-counter drugs for dengue. However, it is important that these preparations are properly standardized and scientifically validated [31]. Though some studies have established that* E. hirta* is less toxic in murine models [32], it is essential to investigate its toxicity in the human context [33].

## 5. Conclusions

According to the present survey, it is reiterated that the* E. hirta* is a potential therapy against dengue as it holds a significant antiviral and platelet increasing activities. However, relatively few studies have been carried out to accept the clinical application of* E. hirta* as an antidengue therapy. To overly validate the traditional claim, more studies on contemporary pharmacological approaches including isolation of active compounds and elucidating the mode of action of antidengue activities are warranted. Further, well-controlled double-blind clinical trials are required to reevaluate the efficacious and side effects in order to establish its place in clinical applications.

## Figures and Tables

**Table 1:** Summary of studies conducted on antidengue activity of *Euphorbia hirta*.

Number	Study	Experimental model	Part of the plant/preparation method	Outcome
1	[19]	Ethnopharmacological survey	Decoction of leaves or bark	Predominately 60–80 years of age mostly females with primary and secondary education were aware of the use of *E. hirta* for dengue.

2	[20]	Descriptive ethnobotanical survey	Expressed juice of *E. hirta*	A survey conducted using questionnaires in Agoo, La Union, Philippines revealed Tawa-Tawa is the most sought medicinal plant for dengue.
		*In vivo* assay on rabbits (Aspirin-induced thrombocytopenia)	Expressed juice of *E. hirta*	A significant increase of platelet count after 24 hours of administration of *E. hirta* juice into thrombocytopenic rabbits.

3	[21]	*In vivo* assay on rats (ethanol (i.p injection) induced thrombocytopenic model)	Decoction of fresh whole plant	A significant increase of platelet counts after 14 days of administration of *E. hirta,* further reporting decreased bleeding time and clotting time of rats.

4	[22]	*In vivo* assay using rats (thrombocytopenia was induced using Anagrelide)	Water extract of leaves	Mean platelet count was increased by 80% following *E. hirta* treatment for 9 days.

5	[23]	Clinical study using dengue patients admitted to Sir Ganga Ram Hospital, Lahore.	Herbal water	Over 70% patients exhibited a platelet increase. Marked recovery in fever and flu like symptoms following 24 hours of administration of *E. hirta.*

6	[25]	*In vitro* assay for DENV-2 serotype	Ethanol extract of leaves	Virus inhibition by 34.7%.

7	[24]	*In vitro* assay	Tea of *E. hirta* and ethyl acetate/methanol and ethyl acetate partitioning.	The ethyl acetate fraction of whole plant of *E. hirta *extracted using methanol and dichloromethane significantly reduced (85%) the plaque forming capacity of dengue virus serotype 1. Nine compounds were isolated from the fraction.

8	[26]	Molecular docking of phytochemicals with 2FOM-dengue proteases, 2P40-methyl transferase of dengue	Leaves of *E. hirta*	Quercetin exhibited strongest binding with dengue virus. Thus, *E. hirta* can be indicated as effective against dengue virus.
